# MAPK/ERK Signaling in Tumorigenesis: mechanisms of growth, invasion, and angiogenesis

**DOI:** 10.17179/excli2025-8479

**Published:** 2025-07-23

**Authors:** Jiaying Fei, Yanjun Guo

**Affiliations:** 1 Department of Human Anatomy, Medical College, Jiaxing University, Jiaxing, Zhejiang, China

**Keywords:** ERK1/2, tumorigenesis, Ras-Raf-MAPK pathway, tumor proliferation, tumor invasion, angiogenesis

## Abstract

The significance of ERK1/2 in the process of tumorigenesis has attracted considerable interest owing to its essential role in a variety of cellular mechanisms, especially in relation to cancer initiation and progression. The Ras-Raf-MAPK signaling cascade, responsible for the activation of ERK1/2, plays a vital role in the regulation of tumor cell growth, invasion, and the formation of new blood vessels. Recent research has underscored the intricate nature of the mechanisms by which ERK1/2 is activated and the subsequent implications for tumor biology, illustrating both the oncogenic capabilities and the therapeutic hurdles linked to the modulation of this pathway. Despite progress in the comprehension of ERK1/2 signaling, numerous challenges persist, including the emergence of resistance to therapies that target this pathway, alongside the necessity for more selective inhibitors. This review intends to consolidate the most recent scientific discoveries pertaining to ERK1/2 and its regulatory influence within the Ras-Raf-MAPK pathway, offering insights into how these interactions facilitate tumor proliferation and metastasis. By clarifying the connection between ERK1/2 signaling and tumor biology, this article aspires to contribute to the formulation of novel therapeutic approaches aimed at interrupting this pathway in the context of cancer treatment.

## Introduction

Recently, the role of Extracellular signal-regulated kinase l and 2 (ERK1/2) in the Ras/Raf/MEK/ERK signaling pathway has received significant attention for its relevance to tumorigenesis (Zhang et al., 2012[148]; Matsushita et al., 2009[91]). ERK1/2 is essential for integrating various signals. It combines extracellular growth factor signals like epidermal growth factor (EGF) and fibroblast growth factor (FGF) with intracellular oncogenic signals caused by mutations in KRAS and BRAF (Clerk et al., 2006[19]; Martini et al., 2013[89]). This combination enables the modulation of essential cellular activities, such as growth, invasion, metastasis, and the formation of new blood vessels, which are crucial for the advancement of cancer (Coura et al., 2019[21]; Ragab et al., 2025[109]). The disruption of this signaling pathway, especially due to mutations such as KRASG12D (Hill et al., 2010[50]; Gurreri et al., 2023[44]), causes continuous activation of ERK1/2, which in turn leads to abnormal expression of cell cycle regulators, including cyclin D1, as well as apoptosis inhibitors like Bcl-2. This ultimately enhances the survival and proliferation of tumor cells (Huang et al., 2023[55]; Zhou et al., 2020[153]).

Furthermore, the interaction between ERK1/2 and other signaling cascades, such as PI3K/AKT and Wnt/β-catenin (Wang et al., 2011[136]; Boo et al., 2013[10]), significantly heightens its oncogenic capabilities. For example, the phosphorylation of glycogen synthase kinase 3 beta (GSK-3β) mediated by ERK leads to the stabilization of β-catenin (Li et al., 2018[66]; Zheng et al., 2013[151]), which is a crucial component in the process of epithelial-mesenchymal transition (EMT), essential for cancer metastasis (Liu et al., 2022[72]). Consequently, the interplay among these signaling pathways not only boosts the proliferative and invasive properties of tumor cells but also complicates treatment strategies that are focused on the ERK1/2 pathway.

Targeting the ERK1/2 signaling cascade has emerged as a promising strategy to counteract resistance mechanisms in RAS-driven tumors (Jameson et al., 2013[59]; Roskoski, 2019[111]). Clinical efforts have focused on the development of inhibitors targeting various components of the pathway, including RAF (e.g., Sorafenib), MEK (e.g., Trametinib), and ERK itself (e.g., Ulixertinib). However, the effectiveness of these therapies is frequently restricted by resistance, particularly in BRAF V600E mutant melanoma (Cintolo et al., 2016[18]; Corazzari et al., 2015[20]), where MEK inhibition can reactivate ERK signaling (Yin et al., 2024[142]).Additionally, the tumor microenvironment, through factors such as interleukin-6 (IL-6), can activate the JAK/STAT pathway (Lesina et al., 2014[65]; Lu et al., 2023[77]), that synergizes with ERK signaling, further diminishing the efficacy of targeted therapies (Ryan et al., 2024[113]).

Recent studies have highlighted the significant roles of epigenetic regulation and metabolic reprogramming in developing resistance to ERK pathway inhibitors, illustrating the complexity of tumor biology. For example, demethylation of the MDR1 promoter and enhanced glycolysis have been implicated in the adaptive responses of tumor cells to ERK pathway inhibition (Huang et al., 2023[55]; Gaghan et al., 2024[33]). Consequently, understanding the diverse roles of ERK1/2 in tumor biology is crucial for creating effective therapeutic strategies.

In conclusion, this review clarifies how ERK1/2 contributes to tumor formation in different types of cancer and highlights its importance as a therapeutic target. We will examine the structural and functional aspects of the Ras-Raf-MAPK pathway to provide insights that enhance our understanding of cancer biology and guide the development of more effective treatments. The following sections will explore the key characteristics of the Ras-Raf-MAPK pathway and its critical role in cancer pathology, setting the stage for potential therapeutic advancements.

## ERK1/2 Structure and Function

### ERK1/2 molecular structure

**Figure 1:**
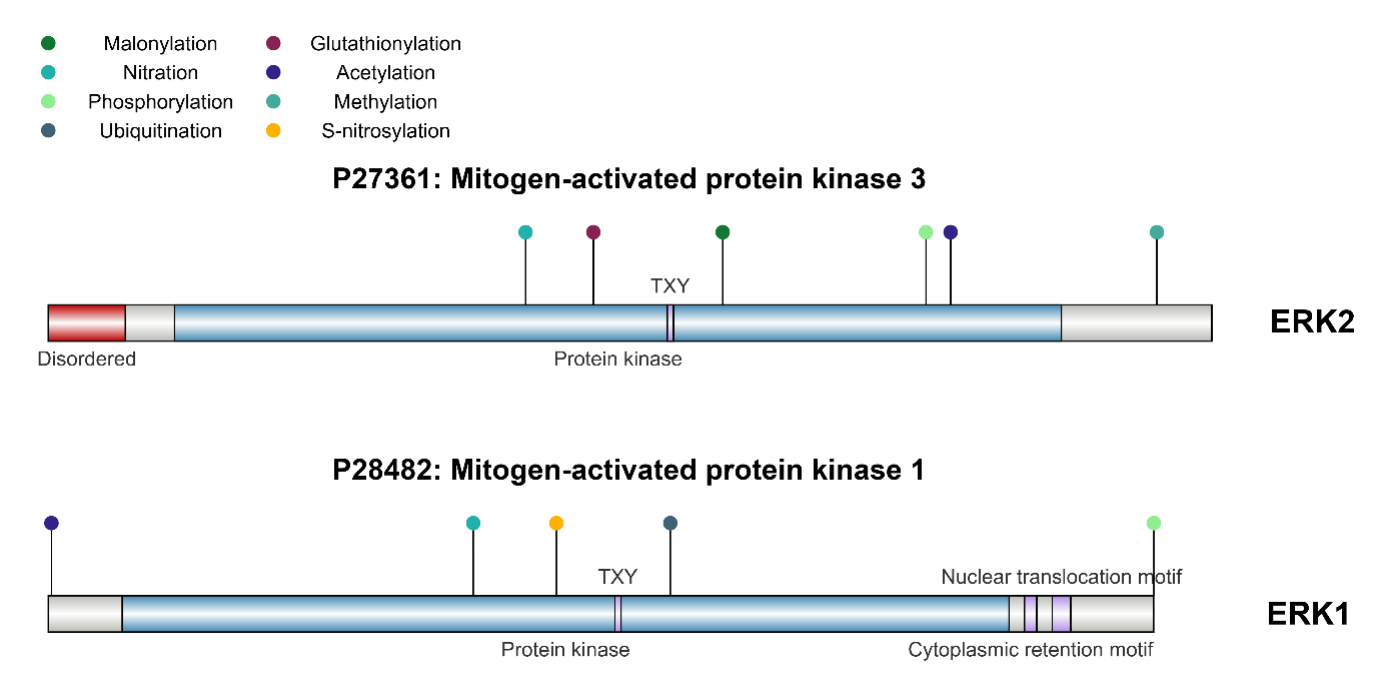
The image illustrates the structure of human ERK1 and ERK2.Full length ERK1/2 amino acid sequences of human were retrieved from the NCBI database.ERK1/2 functional domains were mapped using IBS software and recolored.

Extracellular signal-regulated kinases 1 and 2 (ERK1/2) are pivotal components of the mitogen-activated protein kinase (MAPK) signaling pathway, which plays a critical role in mediating cellular responses to growth factors and other stimuli (Yao et al., 2003[140]; Zhang et al., 2012[148]). ERKl and ERK2 are serine/threonine kinases with a high degree of similarity. ERK2, being the more studied isoform due to its predominant expression invarious tissues. Both ERKl and ERK2 contain a conserved kinase domain, which is crucial for their enzymatic activity, along with a regulatory domain that controls their activation state (Marampon et al., 2019[85]). The activation of ERK1/2 occurs through a dual phosphorylation mechanism on threonine and tyrosine residues within a specific TEY motif, which is crucial for its kinase activity(Arkell et al., 2008[6]). This phosphorylation is carried out by upstream kinases, MEKl and MEK2, which are activated by the RAS/RAF signaling cascade (Roskoski, 2012[110], 2018[112]).The structure of ERK1/2 enables it to interact with various substrates, including transcription factors, cytoskeletal proteins, and other signaling molecules. This interaction influences numerous cellular processes, such as proliferation, differentiation, and apoptosis. Understanding the structure of ERK1/2 is crucial for developing targeted therapies that aim to modulate its activity in cancers where this pathway is abnormally activated (Hossain, 2024[52]) (Figure 1(Fig. 1)).

### Upstream action of Ras proteins

**Figure 2:**
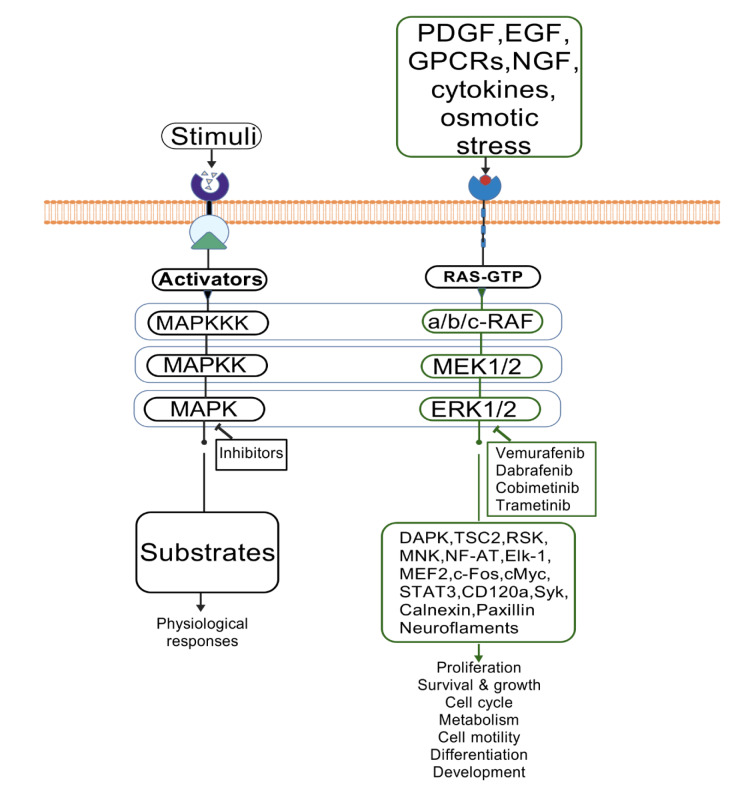
The image illustrates ERK cascades and their physiological functions.MAP kinases, located in the cytoplasm with the ability to translocate into the nucleus, facilitate the phosphorylation of a wide array of cytosolic proteins as well as various nuclear transcription factors. This process results in a multitude of physiological effects, encompassing cellular proliferation, differentiation, and developmental processes.

Ras proteins, such as KRAS, NRAS, and HRAS, are small GTPases that function as molecular switches in the MAPK signaling pathway. They play a crucial role in transmitting signals from different growth factor receptors to downstream effectors. These include RAF kinases, which then activate the MEK/ERK cascade. Ras is activated when growth factors bind to receptor tyrosine kinases (RTKs), causing the exchange of GDP for GTP and converting it to its active form (Markevich et al., 2004[86]).The active Ras-GTP complex interacts with and activates RAF kinases, specifically A-Raf, B-Raf,and C-Raf, which are responsible for phosphorylating MEK1and MEK2 (Roskoski, 2018[112]). Dysregulation of Ras signaling often due to mutations in KRAS, is implicated in several cancers, including pancreatic, colorectal, and lung cancer (Luo, 2021[80]; Mann et al., 2016[84]). These mutations lead to constitutive activation of Ras, resulting in persistent activation of the MAPK pathway, promoting uncontrolled cell proliferation and survival. Understanding the upstream action of Ras is essential for developing targeted therapies that inhibit its activity or downstream signaling components in Ras-driven malignancies (Therachiyil et al., 2022[130]) (Figure 2(Fig. 2)).

### Phosphorylation process of Raf kinases

Raf kinases, especially B-Raf, play a crucial role in the MAPK signaling pathway, functioning downstream of Ras proteins (Hatzivassiliou et al., 2010[48]). Raf phosphorylation is essential for its activation and signaling. When activated by Ras-GTP, Raf changes shape and moves to the plasma membrane, where it interacts with proteins associated with the membrane. This interaction promotes Raf's phosphorylation at specific serine and threonine residues, which is vital for its complete activation. The phosphorylation of B-Raf at serine 445 and threonine 573, among others, enhances its kinase activity and promotes the phosphorylation of MEK1 and MEK2. This dual phosphorylation of MEK is necessary for its activation, which then leads to the phosphorylation of ERK1/2 (Dwivedi et al., 2009[29]; Roskoski, 2019[111]). In cancers where B-Raf is mutated, such as the BRAF V600E mutation, the kinase is constitutively active, resulting in continuous signaling through the MAPK pathway, contributing to tumorigenesis (Bharti et al., 2025[8]; Brady et al., 2014[11]). Thus, focusing on the phosphorylation of Raf kinases offers a promising treatment approach to block abnormal MAPK signaling in cancer (Dillon et al., 2021[24]).

### Downstream signal transduction of ERK1/2

Once activated, ERK1/2 translocate to the nucleus, where they phosphorylate a variety of substrates, including transcription factors such as c-Fos, c-Jun, and Elk-1. This phosphorylation regulates gene expression, which is essential for cell cycle progression, differentiation, and survival. ERK1/2 also influence cytoplasmic processes, including the modulation of cytoskeletal dynamics and the enhancement of cell migration and invasion (Acconcia et al., 2006[2]). The effects of ERK1/2 signaling depend on the context and can vary according to the cellular environment and the specific stimuli. In cancer, aberrant activation of the ERK1/2 pathway often results in enhanced cell proliferation and resistance to apoptosis (Zhang et al., 2020[149]; Zhou et al., 2011[152]), contributing to tumor growth and metastasis. Additionally, feedback mechanisms allow ERK1/2 to phosphorylate upstream components like Raf and Ras. This creates a complex regulatory network that can result in both positive and negative feedback loops. This intricate signaling network underscores the importance of ERK1/2 in maintaining cellular homeostasis and its potential as a therapeutic target in cancer treatment (Liu et al., 2023[75]) (Figure 2(Fig. 2)).

### Core mechanism of Ras/Raf/MAPK pathway

**Figure 3:**
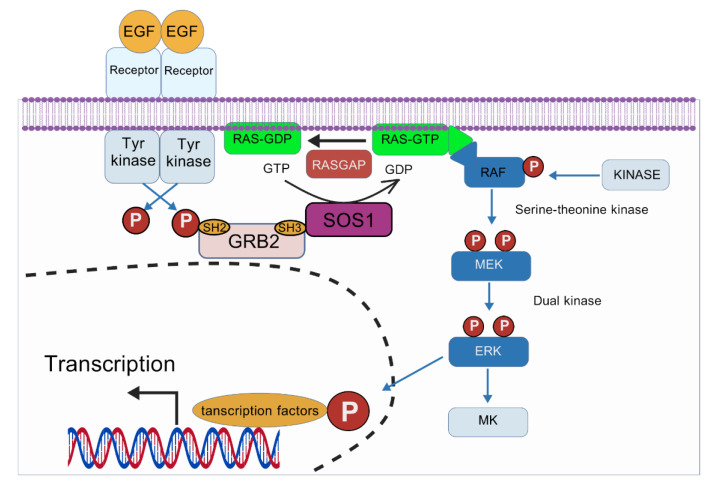
The image illustrates RAS/RAF/MEK/ERK pathway.Membrane-bound GTP-loaded RAS recruits and activates RAF kinases, which phosphorylate MEK1/2. Activated MEK then phosphorylates ERK1/2 on tyrosine and threonine residues. Activated ERK translocates to the nucleus, phosphorylating cytosolic proteins and nuclear transcription factors to regulate cell fate.

The Ras/Raf/MAPK pathway is a key signaling cascade that controls various cellular processes, such as growth, differentiation, and survival (Leicht et al., 2007[64]; Guo et al., 2020[42]). The core mechanism consists of the step-by-step activation of Ras, Raf, MEK, and ERK. When stimulated by growth factors, Ras is activated and binds to Raf. This binding causes Raf to become phosphorylated and activated. Activated Raf then phosphorylates MEK, which in turn activates ERK through dual phosphorylation (Muslin, 2005[95]; Dwivedi et al., 2009[29]). This cascade is carefully controlled by feedback mechanisms and scaffolding proteins, which ensure both specificity and timing in signaling. When this pathway is dysregulated, often due to mutations in Ras or Raf, it can result in oncogenic transformation and is associated with various cancers.. The pathway's role in mediating responses to external stimuli and its involvement in cell fate decisions make it a critical target for therapeutic interventions. Inhibitors targeting different components of this pathway are being developed and tested in clinical settings, highlighting the pathway's significance in cancer therapy (Chen et al., 2024[17]) (Figure 3(Fig. 3)).

### Activation mechanism of ERK1/2 and negative feedback regulation

ERK1/2 activation mainly occurs through the phosphorylation by MEK1 and MEK2, which are activated by Raf kinases (Su et al., 2010[126]). When growth factor receptors send signals, Ras activates Raf, which then phosphorylates MEK and subsequently ERK (McCubrey et al., 2007[93]; Sebolt-Leopold, 2004[116]). This activation is tightly controlled by negative feedback mechanisms that prevent excessive signaling. For example, activated ERK can phosphorylate and inhibit upstream components like Raf and MEK. This creates a feedback loop that limits both the duration and intensity of the signal. Various phosphatases, including dual specificity phosphatases (DUSPs) (Arnoldussen and Saatcioglu, 2009[7]; Li et al., 2021[67]), can dephosphorylate ERK, reverting it to an inactive state. This negative feedback regulation is crucial for maintaining cellular homeostasis and preventing uncontrolled cell proliferation. In cancers where these feedback mechanisms are disrupted (Haney et al., 2016[47]; Hsu et al., 2016[54]), such as through mutations in Ras or Raf, the result is often persistent ERK activation, contributing to tumorigenesis and resistance to therapies. Understanding these regulatory mechanisms is essential for developing effective strategies to target the MAPK pathway in cancer treatment (Hong et al., 2023[51]).

## ERK1/2 Regulation Network in Tumor Proliferation

**Figure 4:**
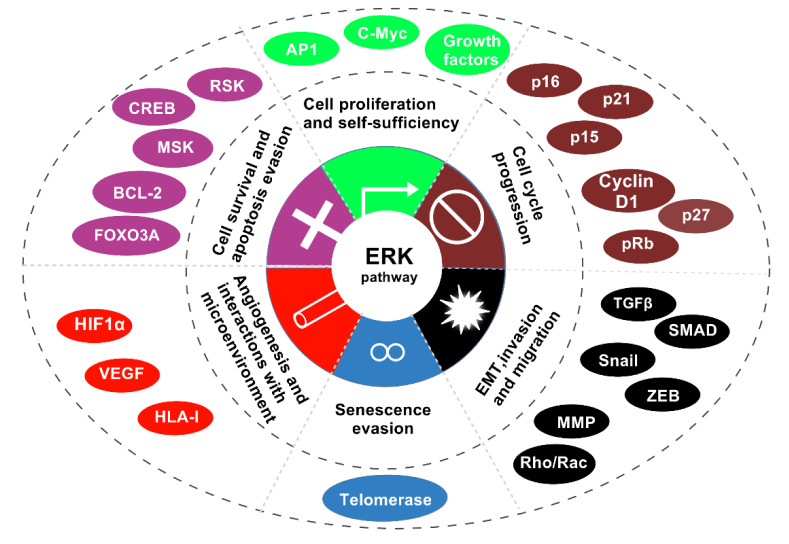
The image illustrates biological consequences of the Ras-ERK pathway activation and the main targets.The six biological effects of Ras-ERK pathway activation and their action targets are cell cycle progression, EMT, invasion and migration, senescence evasion, angiogenesis and interactions with the microenvironment, cell survival and apoptosis evasion, cell proliferation and self-sufficiency.

The ERK1/2 signaling pathway is an essential part of the mitogen-activated protein kinase (MAPK) cascade, which regulates important cellular processes like proliferation, differentiation, and survival. Dysregulation of the ERK1/2 pathway is frequently linked to the progression of various cancers, highlighting its importance in tumor biology. Understanding the ERK1/2 regulatory mechanisms in tumor proliferation is crucial for developing targeted therapies. This section will detail how ERK1/2affects tumor proliferation by examining its role inmodulating growth signals, influencing the cell cycle, and mediating autocrine and paracrine signaling (Figure 4(Fig. 4)).

### Regulation of proliferative signals

The ERK1/2 pathway is primarily activated by growth factors and cytokines that attach to receptor tyrosine kinases (RTKs).This triggers a series of phosphorylation events that activate ERK1/2. This activation is essential for converting external signals into responses that encourage cell growth. For example, in cancers like breast and colorectal cancer, irregularities in the ERK1/2 signaling pathway are associated with increased cell growth and survival. Research indicates that mutations in upstream components of the pathway, such as KRAS and BRAF, cause the continuous activation of ERK1/2, leading to unchecked cell proliferation (Dillon et al., 2021[24]).

Additionally, the interaction between ERK1/2 and other signaling pathways, including the PI3K/AKT pathway, amplifies proliferative signals. For example, the activation of ERK1/2can promote the expression of cyclins and other cell cycle regulators, facilitating the transition from the Gl phase to the S phase of the cell cycle. This mechanism is particularly evident in hepatocellular carcinoma (HCC), where the synergistic interaction between ERK1/2 and Pl3K signaling enhances cell proliferation and survival in response to growth factors (Kim et al., 2019[62]). Furthermore, the activation of ERK1/2is linked to the upregulation of genes involved in metabolic pathways that facilitate rapid cell division. This underscores the significance of this signaling cascade in tumor biology.

### Impact on the cell cycle

The ERK1/2 pathway plays a crucial role in the cell cycle, especially during the change from the G1 phase to the S phase. When ERKl/2 is activated, it phosphorylates several downstream targets, including transcription factors that control the expression of cyclins and cyclin-dependent kinases (CDKs). For instance, the activation of ERK1/2promotes the expression of cyclin Dl, which is critical for the progression of the cell cycle (Huang et al., 2023[55]). In breast cancer, dysregulated ERK1/2 signaling is associated with changes in cell cycle dynamics, which contribute to the aggressive behavior of tumors.

Additionally, ERK1/2 plays a role in both cell cycle regulation and the response to DNA damage. Research shows that ERK1/2 can alter the activity of checkpoint proteins that manage the cell cycle when faced with genotoxic stress. For instance, inhibiting ERK1/2 signaling increases cancer cells' sensitivity to DNA-damaging agents. This indicates that targeting this pathway may enhance the effectiveness of current chemotherapy treatments (Huang et al., 2023[55]). The potential of ERK1/2 as a therapeutic target in cancer treatment is underscored by its dual role in promoting cell cycle progression and participating in DNA damage response mechanisms.

### Autocrine and paracrine mechanisms

The regulation of ERK1/2 signaling is influenced by autocrine and paracrine mechanisms that enable communication between tumor cells and their microenvironment. By secreting growth factors and cytokines, tumor cells activate ERK1/2 signaling not only in themselves but also in neighboring cells. For example, in breast cancer, tumor cells produce factors like IL-6 and CXCL1 that activate ERK1/2 signaling pathways, promoting tumor proliferation and survival (Khojasteh et al., 2021[60]).

Moreover, the interaction between tumor cells and stromal cells, including cancer-associated fibroblasts (CAFs), plays a crucial role in modulating ERK1/2 activity. CAFs secrete various factors that enhance the proliferative signals tumor cells receive. This process promotes tumor growth.. For example, studies have shown that CAF-derived factors can activate ERK1/2 signaling in neighboring tumor cells, leading to increased proliferation and migration (Song et al., 2024[123]). This paracrine signaling not only supports tumor growth but also helps create a tumor-promoting microenvironment.

In conclusion, the ERK1/2 signaling pathway is a key regulator of tumor proliferation. It influences various aspects of cell cycle progression and is modulated by both autocrine and paracrine mechanisms. Understanding these regulatory networks is crucial for developing targeted therapies. These therapies aim to effectively disrupt the dysregulated signaling pathways present in cancer cells. More research is needed to understand the intricate connections between ERK1/2 signaling and other pathways, and to explore how the tumor microenvironment affects these connections.

## ERK1/2 Mediated Tumor Invasion Molecular Mechanisms

### Tissue Remodeling and Cell Migration

The ERKl/2 pathway is crucial for tissue remodeling and cell migration, both of which are essential for tumor invasion and metastasis. ERKl/2 is a key part of the MAPK signaling pathway. This pathway is activated by different growth factors and cytokines. Upon activation, ERK1/2 translocates to the nucleus where it regulates the expression of genes involved in cell proliferation, survival, and migration (Qin et al., 2023[107]). Remodeling of the extracellular matrix (ECM) is essential for tumor cells to migrate and invade surrounding tissues. Matrix metalloproteinases (MMPs), particularly MMP-2 and MMP-9, are crucial in this context as they degrade ECM components, facilitating the movement of cancer cells (Huang et al., 2023[55]). In tumors, MMP expression is often increased, while their activity is tightly controlled by tissue inhibitors of metalloproteinases (TIMPs).The balance between MMPs and TIMPs is critical; an increase in MMP activity relative to TIMP levels leads to enhanced tissue remodeling, which promotes tumor invasion (Yu et al., 2024[143]). Additionally,ERK1/2 signaling regulates MMP expression, linking this pathway's activation to the tumor's invasive potential (Dudka et al., 2022[28]) (Figure 4(Fig. 4)).

Besides regulating matrix metalloproteinases (MMPs), ERK1/2 signaling also affects cytoskeletal dynamics, which are crucial for cell motility. During cell migration, the actin cytoskeleton is significantly remodeled. ERK1/2 activation is linked to changes in actin filament organization, allowing cancer cells to become more migratory (Zhan et al., 2023[147]). The interaction between ERK1/2 and other signaling pathways, such as the Rho family of GTPases, further highlights its role in regulating the migratory capabilities of tumor cells (Huang et al., 2023[55]). The ERK1/2 pathway orchestrates a complex network of signals that facilitate tissue remodeling and cell migration, contributing to the invasive characteristics of tumors.

### Expression of matrix metalloproteinases

Matrix metalloproteinases (MMPs) are a family of zinc-dependent endopeptidases that play a crucial role in the degradation of the extracellular matrix (ECM), a process essential for tumor invasion and metastasis. The elevated levels of various MMPs, particularly MMP-2 and MMP-9, in malignant tumors often indicate a poor prognosis (Huang et al., 2023[55]). The regulation of MMP expression is complex and involves several signaling pathways, one of which is the ERKl/2 pathway. Activation of the ERKl/2 pathway increases MMP expression, which enhances the invasive potential of cancer cells (Huang et al., 2023[55]).

In cancer, MMPs break down ECM components, enabling tumor cells to migrate through the stroma and invade surrounding tissues. For instance, MMP-9 is linked to the invasion of breast and colorectal cancers, with its expression correlating to tumor progression and metastasis (Huang et al., 2023[55]). The activity of MMPs is tightly regulated by their tissue inhibitors (TIMPs), and an imbalance between MMPs and TIMPs can lead to increased tumor aggressiveness. Studies have shown that overexpressed MMPs, especially in the presence of inflammatory cytokines, can create a pro-tumorigenic microenvironment. This environment further promotes invasion and metastasis (Huang et al., 2023[55]).

Additionally, MMP expression is affected by factors such as hypoxia, which is prevalent in solid tumors. Hypoxia can trigger MMP expression through the HIF-1α pathway, which increases tumor invasiveness (Huang et al., 2023[55]). This emphasizes the complex role of MMPs in cancer biology, as they not only aid in ECM remodeling but also help create a supportive microenvironment for tumor progression.

### Cross-talk of signaling pathways

The interaction of various signaling pathways is crucial in cancer biology, especially regarding tumor invasion and metastasis. The ERKl/2 pathway interacts with other pathways, including Pl3K/Akt and Wnt, rather than functioning alone. This interaction modifies how cells respond to external stimuli (Huang et al., 2023[55]).This cross-talk significantly influences tumor cell invasion by integrating signals from multiple sources.

The PI3K/Akt pathway promotes cell survival and growth. Its activation enhances ERK1/2signaling effects on MMP expression and activity. This interaction can result in a more aggressive tumor phenotype, marked by increased migration and invasion. Furthermore, the Wnt signaling pathway, which regulates cell fate and proliferation, also interacts with ERK1/2 signaling, complicating the regulatory networks that influence tumor behavior (Huang et al., 2023[55]).

Additionally, the interaction between these pathways can contribute to therapeutic resistance. For example, tumors with abnormal activation of both the ERK and Pl3K/Akt pathways may not respond well to therapies that target only one of these pathways (Huang et al., 2023[55]). It is vital to understand these interactions to create effective therapies, as targeting multiple pathways might be needed to overcome resistance and improve clinical outcomes in cancer treatment.

In summary, the ERKl/2 pathway is crucial for tumor invasion. It influences tissue remodeling, regulates MMP expression, and interacts with other signaling pathways. The complexity of these interactions highlights the necessity for a thorough understanding of the molecular mechanisms behind cancer progression. This knowledge could lead to the creation of innovative therapeutic approaches.

## ERK1/2 and Its Relationship with Angiogenesis

The ERK1/2 signaling pathway, a component of the mitogen-activated protein kinase (MAPK) cascade, is crucial for cellular processes, such as proliferation, differentiation, and survival. This pathway is especially important for angiogenesis, the process of forming new blood vessels from existing ones. This process is essential for tumor growth and metastasis. ERK1/2 activation is often triggered by growth factors, particularly vascular endothelial growth factor (VEGF), a key driver of angiogenesis. Therefore, understanding the relationship between ERK1/2 and angiogenesis is crucial for developing effective therapies for cancer and other diseases involving abnormal blood vessel growth (Figure 4(Fig. 4)). 

### Regulation of vascular endothelial growth factor (VEGF)

VEGF plays a crucial role in angiogenesis, and its expression is regulated by several signaling pathways, including the ERK1/2 pathway (Liu et al., 2016[71]; Ding et al., 2022[25]). Activation of ERKl/2 by upstream signals like growth factors increases VEGF expression, which promotes the proliferation and migration of endothelial cells. Research indicates that phosphorylating ERK1/2 boosts VEGF expression indifferent cell types, such as endothelial and tumor cells (Shu et al., 2002[118]; Yamada et al., 2015[139]). For example, in hypoxic conditions, which are common in tumors, the ERKl/2 pathway mediates the upregulation of VEGF. This pathway is activated by hypoxia-inducible factor (HlF) (Sutton et al., 2007[128]; Li et al., 2008[68]). This mechanism underscores the role of ERK1/2 in how hypoxic stress affects angiogenesis, helping tumors adapt to low oxygen by promoting new blood vessel formation.

The relationship between VEGF and ERKl/2 is reciprocal: VEGF activates the ERK1/2 pathway, and in turn, ERK1/2 enhances VEGF signaling through several feedback mechanisms. For instance, studies show that when ERKl/2 is activated, it increases the expression of VEGF receptors, which boosts the angiogenic response (Andrikopoulos et al., 2017[5]; Aiken and Birot, 2016[3]). This feedback loop is essential for balancing angiogenesis and vascular stability, particularly in tumor microenvironments where abnormal angiogenesis frequently happens (Gianni-Barrera et al., 2020[38]; Luo et al., 2023[79]). 

### Molecular mechanisms of angiogenesis

The mechanisms of angiogenesis are complex and involve many signaling pathways and cellular interactions. The extracellular signal-regulated kinase 1/2 pathway plays a central role in these processes. It integrates signals from various growth factors and cytokines to regulate the behavior of endothelial cells. When activated, this pathway promotes key processes important for angiogenesis, such as endothelial cell proliferation, migration, and tube formation.

A crucial aspect of angiogenesis is the remodeling of the extracellular matrix (ECM), essential for forming new blood vessels (Sottile, 2004[125]; Bogaczewicz et al., 2006[9]). The ERKl/2 signaling pathway regulates the expression of matrix metalloproteinases (MMPs) that degrade ECM components, allowing endothelial cells to migrate and form new capillary structures. This pathway also regulates cell adhesion molecules, which are crucial for maintaining the stability of endothelial cell junctions during angiogenesis.

Additionally, ERK1/2 plays a role in angiogenesis that extends beyond endothelial cells. It also affects pericytes and smooth muscle cells, which are crucial for stabilizing newly formed blood vessels (Halaidych et al., 2019[45]; Abraham et al., 2008[1]). ERK1/2 regulates the interaction between endothelial cells and these supporting cells, ensuring that the newly formed vessels are functional and stable.

In cancer and other diseases, improper regulation of the ERKl/2 pathway can result in excessive blood vessel formation, which promotes tumor growth and spread. Consequently, researchers are exploring strategies to target this pathway to inhibit angiogenesis in tumors (Qin et al., 2023[108]). Various inhibitors of the MAPK pathway are being tested in clinical trials to reduce tumor blood vessel formation and improve patient outcomes (Podar et al., 2004[103]).

### Prospects of anti-angiogenic therapy

The potential of targeting the ERK1/2pathway for anti-angiogenic therapy is highly promising, particularly due to the crucial role of angiogenesis in tumor progression. Anti-angiogenic strategies today mainly concentrate on blocking VEGF signaling using monoclonal antibodies and small-molecule tyrosine kinase inhibitors (Sia et al., 2014[119]). However, these therapies often face challenges such as drug resistance and limited efficacy.

Combining anti-angiogenic agents with therapies that target the ERK1/2 pathway may improve treatment outcomes. This approach could overcome resistance mechanisms and help normalize tumor blood vessels. For example, studies show that using ERK inhibitors together with VEGF-targeted therapies can more effectively suppress tumor growth and metastasis (Lang et al., 2008[63]; Dai et al., 2009[22]). This combination approach aims to inhibit the formation of new blood vessels and enhance the delivery and effectiveness of concurrent chemotherapy.

Furthermore, discovering biomarkers related to the ERKl/2 pathway activation can assist inidentifying patients who are likely to benefit from anti-angiogenic therapies. Personalized treatment strategies that take into account the tumors' molecular profiles can improve clinical outcomes and reduce toxicity.

In conclusion, understanding the complex relationship between ERK1/2 signaling and angiogenesis is essential for tumor biology and opens avenues for further exploration. Ongoing research into the molecular mechanisms of this relationship will lead to new therapeutic strategies that effectively target angiogenesis in cancer and other diseases with abnormal vascular growth. As our understanding of these pathways deepens, the opportunities for developing more effective anti-angiogenic therapies will continue to grow.

## ERK Pathway and Epigenetic Regulation in Tumorigenesis

**Figure 5:**
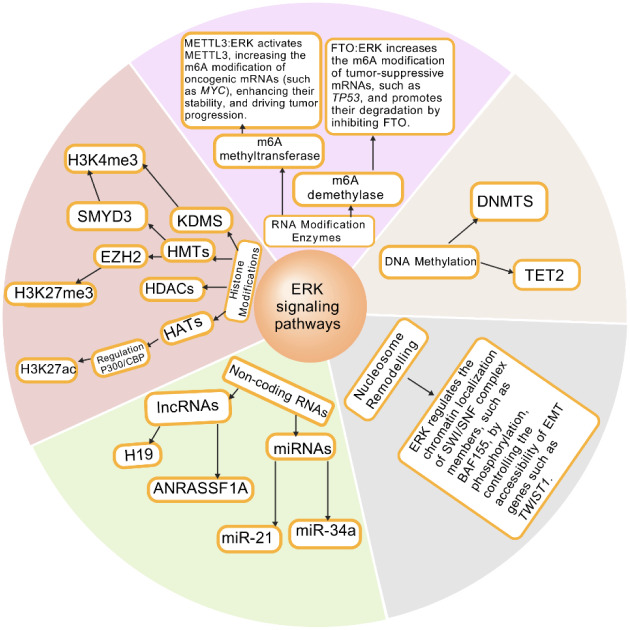
The image illustrates ERK-mediated Epigenetic Regulation in Cancer."In the occurrence of tumors, the ERK pathway and epigenetic regulation include five aspects: DNA methylation, histone modification, RNA modification, nucleosome remodeling, and non-coding RNA.

Recent studies have demonstrated that ERK signaling is influenced by epigenetic mechanisms. Conversely, ERK activation can feedback to modulate the epigenetic landscape, impacting gene expression patterns. Epigenetic regulation encompasses a range of processes that modify gene expression without altering the underlying DNA sequence. These processes include DNA methylation, histone modifications, and the action of non-coding RNAs. The interplay between the ERK signaling pathway and epigenetic mechanisms has garnered significant attention in recent years, as emerging evidence suggests that ERK can influence the activity of epigenetic regulators, while epigenetic changes can also modulate the ERK pathway (Pandian and Ganesan, 2022[100]).For instance, in cancer, aberrant activation of the ERK pathway often leads to uncontrolled cell proliferation and survival, while epigenetic alterations can drive tumorigenesis by silencing tumor suppressor genes or activating oncogenes. Studies have shown that targeting the ERK pathway can have therapeutic benefits in various cancers, but the development of resistance to these therapies remains a significant challenge. Understanding how epigenetic modifications contribute to this resistance could lead to more effective combination therapies that target both the ERK pathway and its epigenetic regulators (Song et al., 2022[124]) (Figure 5(Fig. 5)).

### ERK signaling drives tumorigenesis by regulating DNA methylation

DNA methylation, a crucial epigenetic modification, involves the addition of methyl groups to specific positions on DNA molecules, typically occurring at the 5th carbon position of cytosine (C) residues. The DNA methyltransferase (DNMTs) family plays a central role in this process, primarily including DNMT1, DNMT3A, and DNMT3B. DNMT1 is primarily responsible for maintaining existing DNA methylation patterns, while DNMT3A and DNMT3B are involved in establishing new methylation patterns. Studies have shown that the expression and activity of these enzymes significantly influence the development and progression of various cancers, particularly playing a critical role in the silencing of tumor suppressor genes (Sinclair, 2021[120]). Studies have revealed that the hypermethylation of tumor suppressor genes such as APC, TP53, and SMAD4 in colorectal cancer is closely associated with enhanced tumor aggressiveness and metastatic potential (Nishiki et al., 2025[97]).The ERK pathway induces hypermethylation of tumor suppressor genes (such as tumor suppressor genes and differentiation-related genes) by regulating the activity of DNA methyltransferases (DNMTs). In thyroid cancer, the synergistic interaction between the ERK and PI3K/Akt pathways leads to the epigenetic silencing of genes like PTEN, promoting tumor cell survival and invasion (Gómez Sáez, 2011[41]; Brzezianska and Pastuszak-Lewandoska, 2011[14]). In B-cell lymphoma, DNA hypermethylation in the promoter region of Spry2 (a negative regulator of ERK signaling) results in its transcriptional silencing, thereby relieving inhibition of the MAPK-ERK pathway and enhancing cell proliferation and survival (Frank et al., 2009[32]). This mechanism has been confirmed in both mouse models and human lymphoma, and demethylating drugs (e.g., 5-aza-2'-deoxycytidine) can restore Spry2 expression and suppress ERK activity (Frank et al., 2009[32]). Studies have demonstrated that promoter hypermethylation of RASSF1A is significantly elevated in multiple tumor types, leading to its transcriptional downregulation. This epigenetic silencing further activates the ERK signaling pathway, thereby promoting tumor cell proliferation and metastasis (Mai et al., 2023[83]; Xiong et al., 2025[137]).

### ERK-mediated histone modifications reshape the tumor epigenetic landscape

Histone post-translational modifications represent a pivotal component of epigenetics, regulating gene expression by modulating chromatin structure and transcriptional activity. These modifications include phosphorylation, acetylation, methylation, and ubiquitination. Notably, the acetylation and methylation states of histones are widely recognized as critical regulators of the ERK signaling pathway. During TGF-β-induced epithelial-mesenchymal transition (EMT), rapid ERK activation within 5 minutes is associated with a marked upregulation of histone H3K27 trimethylation (H3K27me3). The methyltransferase Ezh2, responsible for H3K27me3 deposition, synergizes with ERK signaling to promote chromatin condensation and transcriptional activation of EMT-associated genes such as *Snail* and *Twist*, thereby enhancing tumor metastatic potential (Lu et al., 2019[76]).

The ERK signaling pathway dynamically influences gene expression through diverse histone modifications, playing a central role in tumorigenesis. Key mechanisms include: Activating MSK1/2 kinases to induce phosphorylation of histone H3 at serine 10/28 (H3S10ph/S28ph), which facilitates chromatin relaxation and proto-oncogene transcription (McCoy et al., 2020[92]; Zhang et al., 2019[150]; Park et al., 2021[101]); Enhancing p300/CBP-mediated acetylation of histone H3 at lysine 9/27 (H3K9ac/K27ac) to drive pro-tumorigenic gene expression (Fang et al., 2022[30]; Li et al., 2018[70]; Gupta et al., 2020[43]); Phosphorylating SMYD3 to elevate H3K4 trimethylation (H3K4me3), counteracting DNA methylation-mediated silencing of tumor suppressor genes (Hamamoto et al., 2021[46]; Wang et al., 2019[134]); Suppressing EZH2 activity to reduce H3K27me3 levels, thereby alleviating repression of differentiation-associated genes (Kim et al., 2020[61]; Suva et al., 2021[129]); Upregulating RNF20/40 via ELK1 to promote H2B ubiquitination at lysine 120 (H2BK120ub), sustaining DNA repair capacity (Nakamura et al..2022[96]; Chen et al., 2023[16]). These modifications cooperatively regulate cell proliferation, invasion, and chemoresistance through a "histone code" mechanism. Targeting the ERK-epigenetic crosstalk-via strategies such as combined MSK and HDAC inhibitors-has emerged as a promising therapeutic approach in oncology (Zhang et al., 2019[150]; Wang et al., 2019[134]).

### ERK signaling and epigenetic non-coding RNAs

The interplay between ERK signaling and epigenetic non-coding RNAs (ncRNAs) constitutes a sophisticated regulatory axis that amplifies oncogenic programs through bidirectional crosstalk. ERK activation dynamically modulates the expression of long non-coding RNAs (lncRNAs) and microRNAs (miRNAs) via phosphorylation of transcription factors (e.g., c-Myc, AP-1) or chromatin-modifying enzymes.The bidirectional regulatory interplay between the ERK signaling pathway and non-coding RNAs (ncRNAs) plays a pivotal role in cellular differentiation, cancer progression, drug response, and disease pathogenesis.

ERK-Mediated Regulation of ncRNAs: In hepatocellular carcinoma (HCC), hyperactivation of the BRAF/MEK/ERK pathway drives tumor proliferation and drug resistance by modulating lncRNAs. For instance, hypoxia-induced upregulation of the lncRNA *H19* enhances P-glycoprotein expression via ERK signaling, thereby promoting chemotherapeutic drug efflux and resistance (Li et al., 2015[69]; Sokolov et al., 2024[122]; Gnoni et al., 2019[39]).

ncRNA-Mediated Feedback Control of ERK Signaling: FOXM1, a downstream effector of ERK, is post-transcriptionally regulated by miRNAs and lncRNAs. Certain lncRNAs (e.g., *FOXM1-AS*) function as miRNA sponges to relieve FOXM1 suppression, amplifying ERK activity and accelerating cell cycle progression and metastasis in HCC (Gao et al., 2025[34]). In parathyroid tumors, promoter methylation-induced silencing of the *RASSF1A* gene elevates ERK phosphorylation (pERK/ERK) to drive tumorigenesis, a process potentially linked to dysregulated expression of the lncRNA *ANRASSF1A *(Verdelli et al., 2025[131]).

Therapeutic Implications: Resistance to ERK-targeted therapies (e.g., sorafenib in HCC) is closely associated with lncRNA-mediated epigenetic escape mechanisms. Combinatorial strategies-such as co-inhibiting ERK and oncogenic lncRNAs (e.g., *H19* or *ANRASSF1A*)-show promise in overcoming drug resistance (Sokolov et al., 2024[122]; Verdelli et al., 2025[131]). Emerging evidence highlights natural compounds as dual-target modulators that disrupt m6A modification enzymes (e.g., METTL3, FTO), destabilizing ERK-associated ncRNAs to exert anticancer effects. These findings underscore the potential for developing epigenetic-signaling dual inhibitors (Song et al., 2022[124]; Garcia-Lezana et al., 2021[36]).

## Clinical Research on ERK1/2 and Its Therapeutic Potential

**Figure 6:**
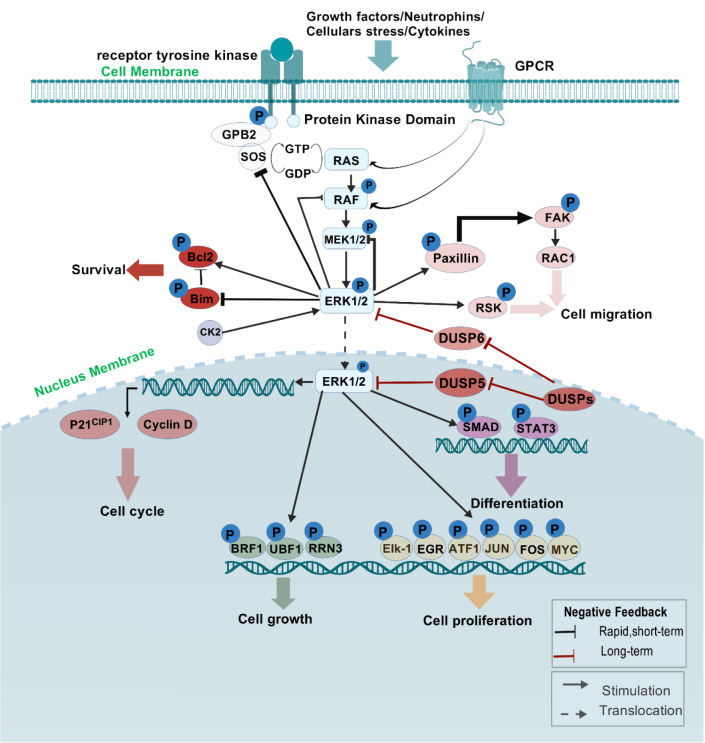
The image illustrates the central position of ERK in signal transduction.Upon the reception of extracellular excitatory stimuli, the Ras/Raf/MEK/ERK signaling pathway is initiated, characterized by a sequential three-tiered phosphorylation cascade that begins at the cellular membrane. The activated ERK1/2 subsequently phosphorylates an extensive array of substrates located within the cellular membrane, cytoskeleton, cytoplasm, and nucleus, thereby facilitating critical cellular processes.

The ERKl/2 signaling pathway is a vital component of the mitogen-activated protein kinase(MAPK) cascade. It plays a significant role in regulating various cellular processes, including proliferation, differentiation, and survival. Because of its role in tumor development, ERKl/2 has become a target for new cancer treatments. Clinical research has focused on developing new inhibitors for ERK1/2, exploring combination therapies, and using biomarkers to improve treatment effectiveness and patient outcomes (Figure 6(Fig. 6)).

### Development of novel inhibitors

The search for effective ERK1/2 inhibitors has intensified, especially in cancers with abnormal MAPK signaling. Recent studies have focused on creating small molecule inhibitors that specifically target the ERK1/2 pathway. For example, Ulixertinib, an established ERK2 inhibitor, has been modified to identify new structures with similar binding properties, showing promising pharmacodynamic and pharmacokinetic profiles in preclinical studies (Pathania et al., 2022[102]). Additionally, new RAF dimer inhibitors like lifirafenib have demonstrated synergistic effects when used with MEK inhibitors. This combination enhances antitumor activity specifically in KRAS-mutant tumors (Yuan et al., 2020[144]). These advancements underscore the therapeutic potential of targeting the ERK1/2 pathway, particularly in cancers with specific genetic alterations.

Ongoing trials are evaluating the clinical efficacy of these inhibitors, focusing on their safety profiles and effectiveness in different types of cancer. For instance, researchers are exploring the combination of ERK inhibitors with standard chemotherapy or immunotherapy. This approach aims to overcome resistance mechanisms and improve patient outcomes. Identifying patients most likely to benefit from these targeted therapies is crucial and requires integrating biomarker-based approaches into clinical trial designs.

### Exploration of combination therapies

Combination therapies have emerged as a promising strategy to enhance the therapeutic efficacy of ERK1/2 inhibitors. The rationale for this approach is based on the complex interactions of signaling pathways involved in tumor progression and resistance, which necessitates the exploration of combination therapies. For example, research shows that combining ERK inhibitors with drugs that target the Pl3K/AKT/mTOR pathway can improve antitumor effects in preclinical models of hepatocellular carcinoma (HCC) (Kim et al., 2019[62]).This combination not only inhibits tumor cell growth but also promotes apoptosis, emphasizing the potential for synergistic interactions between various treatment approaches..

The combination of ERK inhibitors with immune checkpoint inhibitors is currently being investigated to exploit the immunogenic potential of tumors. These combinations may improve overall response rates in patients with advanced malignancies by modifying the tumor microenvironment and boosting T-cell responses. Clinical trials are currently evaluating the safety and efficacy of these combinations. Preliminary results show promising outcomes in certain patient populations (Bratu et al., 2021[12]).

Novel delivery systems, such as nanoparticles, are an important aspect of exploring combination therapies, as they can enhance the bioavailability and targeting of ERK inhibitors. For example, antibody-modified nanoparticles can deliver ERK inhibitors directly to tumor cells, reducing off-target effects and enhancing therapeutic outcomes (Shen et al., 2020[117]). This innovative method highlights the importance of ongoing research to optimize combination therapies and delivery mechanisms for maximizing the clinical benefits of targeting the ERK1/2 pathway.

### Application of biomarkers

The use of biomarkers in ERK1/2-targeted therapies is vital for identifying patients who are most likely to benefit from these treatments. By providing insights into the molecular mechanisms that drive tumorigenesis, biomarkers facilitate the stratification of patients according to their likelihood of responding to therapy. For example, KRAS mutations are common in several types of cancer and are linked to the activation of the MAPK pathway, making them potential biomarkers for selecting patients for ERK1/2-targeted therapies (Hong et al., 2023[51]).

Moreover, downstream effectors of theERK1/2 pathway, like phosphorylated ERK, may act as predictive biomarkers for treatment response. Studies indicate that tumors with high phosphorylated ERK levels tend to be sensitive to ERK inhibitors. In contrast, tumors with low phosphorylated ERK expression may exhibit resistance (Huang et al., 2023[55]). Therefore, it is crucial to develop robust biomarker assays that can reliably assess the activation status of the ERK1/2 pathway in tumor samples.

Liquid biopsies are emerging as non-invasive methods that analyze circulating tumor DNA(ctDNA) and circulating tumor cells (CTCs) to monitor treatment responses and detect resistance mechanisms in real-time. By integrating these technologies with biomarker analysis, we can gain a comprehensive understanding of tumor dynamics and enhance personalized treatment strategies (Moon et al., 2025[94]).

In summary, the rapidly evolving research on ERK1/2 shows great promise, highlighted by advancements in novel inhibitors, combination therapies, and biomarkers that could significantly enhance cancer treatment outcomes. By focusing on these areas, researchers aim to improve treatment outcomes for patients with malignancies linked to aberrant MAPK signaling. Ongoing research is crucial to fully harness the therapeutic potential of targeting the ERK1/2pathway and to develop effective, biomarker-driven strategies for cancer treatment.

## Targeting the Ras/Raf/MAPK Pathway: Therapeutic Strategies

The Ras/Raf/MAPK signaling pathway is crucial for cellular processes like proliferation, differentiation, and survival, making it an important target for cancer therapy. Abnormal activation of this pathway is often seen in different types of cancer, such as melanoma, colorectal cancer, and non-small cell lung cancer (NSCLC) (Poulikakos et al., 2022[105]; Dankner et al., 2018[23]). Targeting this complex signaling cascade, which has many layers of regulation and feedback mechanisms, requires a multifaceted approach. Recent insights into the molecular mechanisms of Ras/Raf/MAPK signaling have led to the development of several therapeutic strategies to inhibit this pathway (Yurugi et al., 2017[145]). These strategies encompass small-molecule inhibitors, monoclonal antibodies, and combination therapies that aim to overcome resistance mechanisms, which often reduce the effectiveness of single-agent treatments (Browne et al., 2009[13]; Sathornsumetee, 2011[114]).

A promising approach is to use selective inhibitors that target specific components of the Ras/Raf/MAPK pathway. For example, MEK inhibitors like trametinib and cobimetinib effectively treat BRAF-mutant melanomas. They work by blocking MEK activation, which is essential for activating ERK1/2 downstream (Alexandraki et al., 2019[4]).These inhibitors provide significant clinical benefits, especially for patients with BRAF V600E mutations, resulting in improved progression-free survival rates (Zeng et al., 2023[146]; Subbiah et al., 2018[127]). However, resistance to MEK inhibitors poses a significant challenge, often arising from feedback activation of the pathway or compensatory signaling through alternative pathways like the Pl3K/Akt/mTOR pathway (Soares et al., 2015[121]; Fourneaux et al., 2017[31]). This highlights the need for combination therapies that can target multiple points within the signaling network at the same time.

Combination therapies strategically enhance the efficacy of Ras/Raf/MAPK pathway inhibitors. For example, combining MEK inhibitors with Pl3K inhibitors has shown synergistic effects in preclinical models, resulting in increased antitumor activity and reduced tumor growth. Additionally, dual-targeting strategies that inhibit both MEK and ERK have emerged as a promising approach to overcoming drug resistance (Goetz et al., 2014[40]; Jaiswal et al., 2018[58]). Recent studies highlight the potential of ERK inhibitors, which block the MAPK pathway downstream of MEK and help circumvent resistance mechanisms linked to upstream inhibitors. The selective ERK1/2 inhibitor, LY3214996 (Ma et al., 2021[82]; Bumrungsup and Kanitpong, 2022[15]), shows promise in preclinical studies. It is currently being evaluated in clinical trials for various malignancies.

Another therapeutic strategy employs monoclonal antibodies targeting receptor tyrosine kinases (RTKs) that activate the Ras/Raf/MAPK pathway. Cetuximab and panitumumab, which target the epidermal growth factor receptor (EGFR), are used to treat colorectal cancer and head and neck cancers (Markman et al., 2009[87]; Martinelli et al., 2009[88]). These antibodies block the activation of downstream signaling pathways, such as the Ras/Raf/MAPK pathway. This action reduces tumor cell proliferation and induces apoptosis (Liu et al., 2022[74]). However, KRAS mutations in tumors often reduce the effectiveness of EGFR-targeted therapies (He et al., 2019[49]; Gattenlöhner et al., 2009[37]), highlighting the need for biomarker-driven methods to identify patients who will most benefit from these treatments.

Researchers are also looking into new therapeutic agents like immunotherapies and targeted nanomedicines to improve targeting of the Ras/Raf/MAPKpathway. For example, using nanoparticles to deliver chemotherapy directly to tumor cells can make the drugs more effective and reduce side effects (Du et al., 2015[26]; Yadav et al., 2021[138]). Moreover, combining immunotherapies with MAPK pathway inhibitors can strengthen antitumor responses by changing the tumor microenvironment and encouraging the immune system to destroy tumor cells.

Although there have been advancements in targeting the Ras/Raf/MAPK pathway, challenges still exist in managing cancers that are driven by abnormal signaling through this pathway. The diversity of tumors and the presence of multiple mutations make treatment strategies difficult, which calls for a personalized approach to therapy (Isaak et al., 2024[56]; Ivanov et al., 2023[57]). Ongoing clinical trials are investigating the efficacy of combination therapies and new therapeutic agents in patients with RAS/RAF mutations, aiming to improve treatment outcomes and decrease the risk of resistance.

In conclusion, targeting the Ras/Raf/MAPK pathway is a promising approach in cancer therapy, offering various options to inhibit this crucial signaling cascade. Selective inhibitors, combination therapies, and innovative delivery methods have significant potential to enhance patient outcomes in malignancies with aberrant MAPK signaling. Ongoing research into resistance mechanisms and the development of new therapeutic strategies will be critical for improving treatments for patients with RAS/RAF-driven cancers.

## Mechanisms and Challenges of Drug Resistance

Drug resistance in cancer therapy is a major obstacle to achieving successful treatment outcomes. Cancer biology is complex due to genetic diversity, adaptive responses, and a changing tumor microenvironment. These factors lead to drug resistant phenotypes, so understanding the mechanisms of drug resistance is essential for developing effective treatment strategies. This section examines the different mechanisms of resistance to cancer drugs and the challenges they create, supported by recent research findings.

A major cause of drug resistance is genetic mutations that change the drug target, making standard therapies ineffective. For example, mutations in the *KRAS* gene, which is crucial in the RAS/RAF/MAPK signaling pathway, are common in several cancers, such as colorectal cancer and non-small cell lung cancer (NSCLC). These mutations result in the continuous activation of downstream signaling pathways, which promotes cell proliferation and survival, even in the presence of targeted therapies (Hong et al., 2023[51]). Moreover, mutations in other parts of the signaling cascade, like BRAF, can also lead to resistance by reactivating the MAPK pathway, even when inhibitors are present (Ma et al., 2021[82]).

Another important factor in drug resistance is tumor heterogeneity. Tumors consist of various cell types, some of which have mutations that make them resistant to certain therapies. This heterogeneity complicates treatment strategies since a single therapy may only work for a subset of tumor cells. For example, in breast cancer, the presence of various subtypes, each with distinct molecular profiles, necessitates tailored treatment regimens to overcome resistance (Ye et al., 2023[141]). Additionally, the tumor microenvironment greatly influences how tumors respond to therapy. Factors such as hypoxia, nutrient deprivation, and the presence of stromal cells can influence drug efficacy and contribute to the development of resistance mechanisms (Ma et al., 2024[81]).

Besides genetic changes and tumor diversity, cancer cells can also become resistant through epigenetic modifications. DNA methylation and histone modification changes can alter gene expression patterns. This alteration promotes cancer cell survival and growth when therapeutic agents are present. For example, improper regulation of genes that control cell death and drug processing can help cancer cells survive treatment (Wang et al., 2024[135]). Additionally, non-coding RNAs, like microRNAs, have been recognized for their role in influencing drug resistance. These small RNA molecules can control the expression of genes related to drug response, which affects how sensitive cancer cells are to treatment (Ye et al., 2023[141]).

The emergence of multidrug resistance(MDR) poses a major challenge in cancer treatment. MDR can occur through various mechanisms. One key mechanism is the overexpression of ATP-binding cassette (ABC) transporters, which actively pump medications out of cancer cells. This reduces the drugs' intracellular concentrations and effectiveness (Duan et al., 2023[27]). In addition, changes in drug uptake mechanisms, increased DNA repair capabilities, and alterations in apoptotic pathways can further contribute to multidrug resistance (MDR) (Vijayakumar et al., 2024[132]). The interaction among these mechanisms creates a complex environment that complicates the treatment of resistant tumors.

Researchers are investigating new therapeutic strategies to tackle drug resistance challenges. Combination therapies target multiple pathways at the same time and have shown promise in overcoming resistance. For example, combining MEK inhibitors with other targeted agents has improved efficacy in KRAS-mutant tumors. This approach prevents the MAPK pathway from reactivating (Hou et al., 2024[53]). Additionally, nanotechnology can improve drug delivery and targeting, thereby reducing off target effects (Lu et al., 2024[78]).

Additionally, precision medicine provides new strategies for addressing drug resistance. By identifying specific genetic alterations in tumors, clinicians can tailor treatment approaches to target the unique vulnerabilities of individual patients. This personalized approach could enhance treatment outcomes and lower the chances of developing resistance (Sattler et al., 2023[115]). Nonetheless, implementing precision medicine presents challenges, such as the necessity for comprehensive genomic profiling and its associated costs.

In conclusion, drug resistance poses a significant challenge in cancer therapy. lt is driven by various mechanisms, including genetic mutations, tumor heterogeneity, epigenetic modifications, and multidrug resistance. It is essential to understand these mechanisms to develop effective treatment strategies. Current research on combination therapies, precision medicine, and new drug delivery systems shows promise for overcoming resistance and enhancing patient outcomes. As our understanding of drug resistance deepens, our strategies for addressing this major barrier in cancer treatment will also evolve.

## Future Directions and Perspectives

The future of cancer therapy, especially in targeting the RAS/RAF/MEK/ERK signaling pathway, looks very promising due to ongoing research and technological advancements in this field. The RAS/RAF/MEK/ERK pathway plays a crucial role in the progression of several cancers, such as colorectal cancer, melanoma, and lung cancer. As researchers explore the complexities of this signaling cascade, new directions are emerging that could greatly improve therapeutic strategies.

A promising approach is developing selective inhibitors that target specific parts of the RAS, RAF/MEK/ERK pathway. Recent studies indicate that inhibitingERK1/2, whether alone or alongside other targeted therapies, significantly reduces tumor growth and metastasis (Pan et al., 2023[99]). For example, the selective ERK1/2inhibitor ASN007 has shown strong antiproliferative effects in RAS/RAF-driven tumors. This suggests that optimizing these inhibitors further could enhance patient outcomes (Portelinha et al., 2021[104]). Moreover, exploring combination therapies that include ERK inhibitors along with other treatments, such as immune checkpoint inhibitors or chemotherapeutics, may improve the overall effectiveness of treatment plans (Gao et al., 2020[35]).

Another important focus is understanding how tumors develop resistance mechanisms in response to targeted therapies. Acquired resistance, especially in RAS-mutant cancers, presents a major challenge in clinical settings. Research shows that the MAPK pathway can be reactivated after initial treatment, resulting in tumor regrowth (Qin et al., 2023[107]). Future studies should focus on identifying biomarkers that predict resistance and developing strategies to overcome it. This may include using dual inhibitors targeting both upstream and downstream components of the signaling pathway (Martin-Vega et al., 2023[90]).

Additionally, the tumor microenvironment's influence on the RAS/RAF/MEK/ERK pathway needs further study. The interaction between cancer cells and their surrounding stroma significantly affects tumor behavior. It also influences how tumors respond to therapy. Understanding these interactions may help identify new therapeutic targets in the microenvironment to enhance treatment efficacy (Wang et al., 2021[133]). For instance, targeting integrins that mediate cell-matrix interactions has shown promise in preclinical models. This suggests that a comprehensive approach, which accounts for the tumor microenvironment, could lead to improved therapeutic outcomes (Liu et al., 2024[73]).

Along with pharmacological advancements, new technologies like CRlSPR/Cas9 gene editing and advanced imaging can enhance the detailed study of the RAS/RAF/MEK/ERK pathway. By enabling researchers to manipulate specific genes within this pathway, these technologies deepen our understanding of the genes' roles in cancer progression and treatment response (Puszkiel et al., 2023[106]). Additionally, using artificial intelligence and machine learning to analyze large genomic datasets may help identify new therapeutic targets and predict how patients respond to specific treatments (Ma et al., 2024[81]).

Finally, personalized medicine is crucial for effectively targeting the RAS/RAF/MEK/ERK pathway, especially as we learn to tailor therapies to individual tumor profiles. As we enhance our understanding of the genetic and molecular foundations of individual tumors, tailoring therapies to a patient's unique tumor profile will become more feasible. This approach could maximize therapeutic efficacy while minimizing adverse effects, ultimately leading to improved patient outcomes and quality of life (Oda et al., 2021[98]).

In conclusion, the future of targeting the RAS/RAF/MEK/ERK signaling pathway in cancer therapy looks promising due to ongoing research and technological innovations. By focusing on selective inhibitors and understanding resistance mechanisms, as well as exploring the tumor microenvironment and leveraging novel technologies, we can pave the way for more effective, personalized cancer treatments that address the complexities of this challenging disease.

## Conclusion

Recent research has enhanced our understanding of the Ras-Raf-MAPK pathway, particularly ERK1/2's role in tumor development, growth, invasion, and angiogenesis. ERK1/2 regulates gene expression and survival pathways critical for cancer, with its dysregulation linked to poor outcomes. Understanding ERK1/2's mechanisms in the tumor microenvironment is essential for targeted therapies. While some studies emphasize ERK1/2's oncogenic potential, others suggest it may also suppress tumors, highlighting the complexity of cancer biology. Developing ERK1/2-targeted therapies could transform cancer treatment, though challenges like resistance need addressing. Interdisciplinary research is vital for advancing our understanding and clinical applications of ERK1/2. Ultimately, effective ERK1/2-targeted therapies could significantly improve cancer treatment outcomes.

## Declaration

### Consent for publication

All authors provided permission for publication.

### Competing interests

No authors have any conflicts of interest or competing interests to declare.

### Funding

This research was funded by the Natural Science Foundation of Zhejiang Province(LGD21H160003).

### Statement on Artificial Intelligence

The authors confirm that artificial intelligence tools were not used in the preparation or analysis of this manuscript. The authors used DeepSeek to check for grammar and style.
